# Negative symptoms in first episode schizophrenia: Results from the “parma early psychosis” program

**DOI:** 10.1192/j.eurpsy.2021.448

**Published:** 2021-08-13

**Authors:** L. Pelizza, E. Leuci, D. Maestri, E. Quattrone, G. Paulillo, P. Pellegrini, S. Azzali

**Affiliations:** 1 Department Of Mental Health, Azienda USL di Parma, Parma, Italy; 2 Department Of Mental Health, Azienda USL-IRCCS di Reggio Emilia, Reggio Emilia, Italy

**Keywords:** psychopathology, negative symptoms, schizophrénia, early psychosis

## Abstract

**Introduction:**

Identifying distinct dimensions of negative symptoms in First Episode Schizophrenia (FES) might result in a better understanding and treatment of this invalidating symptomatology.

**Objectives:**

Aim of this study was to examine negative symptom structure in FES patients using the Positive and Negative Syndrome Scale (PANSS).

**Methods:**

All 147 participants, aged 12–35 years, completed the PANSS and the Global Assessment of Functioning (GAF) scale. A principal component analysis with varimax rotation was performed to investigate PANSS negative symptom structure in the FES total sample.

**Results:**

A 2-factor model (i.e. “Expressive Deficits” and “Asociality” dimensions) was identified. Only “Expressive Deficits” domain had a significant negative correlation with baseline GAF score.

**Conclusions:**

This bipartite solution seems to be adequate to describe the phenomenological variety of negative symptoms experienced by FES individuals at the point of entry in early intervention services.

**Disclosure:**

No significant relationships.

